# Brain mineralocorticoid receptor in health and disease: From molecular signalling to cognitive and emotional function

**DOI:** 10.1111/bph.15835

**Published:** 2022-04-07

**Authors:** Susana N. Paul, Katja Wingenfeld, Christian Otte, Onno C. Meijer

**Affiliations:** ^1^ Department of Medicine, Division of Endocrinology Leiden University Medical Center Leiden The Netherlands; ^2^ Einthoven Laboratory for Experimental Vascular Medicine Leiden University Medical Center Leiden The Netherlands; ^3^ Klinik für Psychiatrie und Psychotherapie Charité Universitätsmedizin Campus Benjamin Franklin Berlin Germany; ^4^ NeuroCure Cluster of Excellence Charité Universitätsmedizin Berlin Berlin Germany

**Keywords:** adaptation, affective state, brain, cognitive and emotional function, glucocorticoids, mineralocorticoids, neural circuits, nuclear receptor, stress

## Abstract

Brain mineralocorticoid receptors (MR) mediate effects of glucocorticoid hormones in stress adaptation, as well as the effects of aldosterone itself in relation to salt homeostasis. Brain stem MRs respond to aldosterone, whereas forebrain MRs mediate rapid and delayed glucocorticoid effects in conjunction with the glucocorticoid receptor (GR). MR‐mediated effects depend on age, gender, genetic variations, and environmental influences. Disturbed MR activity through chronic stress, certain (endocrine) diseases or during glucocorticoid therapy can cause deleterious effects on affective state, cognitive and behavioural function in susceptible individuals. Considering the important role MR plays in cognition and emotional function in health and disease, MR modulation by pharmacological intervention could relieve stress‐ and endocrine‐related symptoms. Here, we discuss recent pharmacological interventions in the clinic and genetic developments in the molecular underpinnings of MR signalling. Further understanding of MR‐dependent pathways may help to improve psychiatric symptoms in a diversity of settings.

**LINKED ARTICLES:**

This article is part of a themed issue on Emerging Fields for Therapeutic Targeting of the Aldosterone‐Mineralocorticoid Receptor Signaling Pathway. To view the other articles in this section visit http://onlinelibrary.wiley.com/doi/10.1111/bph.v179.13/issuetoc

**Abbreviations**
AHN: adult hippocampal neurogenesis
AI: adrenal insufficiency
BPD: bipolar disorder
CA1: cornu ammonis 1
CA2: cornu ammonis 2
CORT: cortisol/corticosterone
CS: Cushing's syndrome
DBD: DNA binding domain
DEX: dexamethasone
GC: glucocorticoid
GR: glucocorticoid receptor
HPA: hypothalamic–pituitary–adrenal (HPA) axis
MDD: major depressive disorder
MR: mineralocorticoid receptor
MRA: mineralocorticoid receptor antagonist
PA: primary aldosteronism
RIME: rapid immunoprecipitation of endogenous proteins

## 1 INTRODUCTION

Corticosteroid hormones affect the brain via the mineralocorticoid receptor (MR) and the glucocorticoid receptor (GR), thus controlling development, metabolism, homeostasis, cognition, and mood. Both types of corticosteroid receptors can exert rapid non‐genomic effects on neuronal activity, but they are better known as ligand‐activated transcription factors that regulate gene transcription. The high‐affinity MR, which was originally termed the type 1 corticosteroid receptor, is encoded by the *NR3C2* gene, whereas the low‐affinity GR, originally termed the type 2 corticosteroid receptor, is encoded by the *NR3C1* gene. Both receptors, with differing affinities to corticosteroids and specific expression patterns throughout the brain, can bind to the same regulatory target sequences of genes. They also control distinct transcriptional networks, to differentially regulate broadly‐defined neuronal processes that range from cell differentiation and survival to cognitive and emotional functions (de Kloet et al., 2005).

**FIGURE 1:**
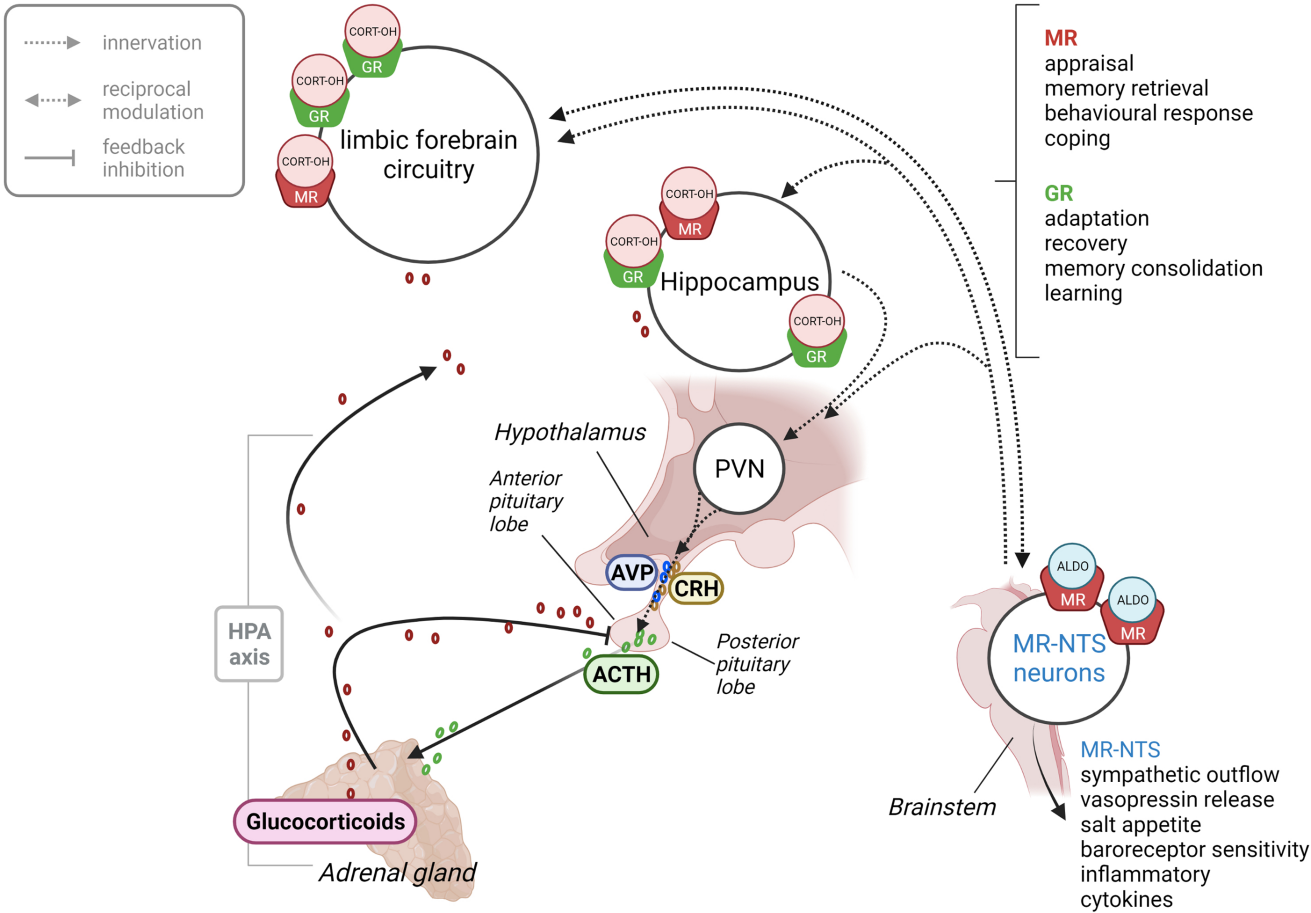
The HPA axis: Corticotropin‐releasing hormone (CRH) is secreted from the paraventricular nucleus (PVN). This hormone in turn stimulates the secretion of ACTH from the anterior pituitary which, in turn drives the secretion of glucocorticoids (cortisol or corticosterone) from the adrenal cortex. Magnocellular neurons of the PVN project directly to the posterior pituitary to release vasopressin (and oxytocin) into the systemic circulation. Aldosterone‐selective MRs, which mediate behaviours involved in salt appetite, are located mostly in neurons of the nucleus tractus solitarii (NTS) and by the circumventricular organs. The MR‐NTS neurons innervate areas of limbic forebrain regions, notably the hippocampus and locus coeruleus areas, which are involved in arousal, and where they reciprocally modulate pathways involved in emotions, memory performance, and reward processing

Corticosteroids include both glucocorticoids, cortisol and corticosterone, and mineralocorticoids such as aldosterone and the less potent 11‐deoxycorticosterone (DOC). In the brain, the main ligands for both MR and GR are glucocorticoids. They are synthesised and released in a circadian, and ultradian, rhythm from the zona fasciculata of the adrenal cortex. Their release is regulated by the hypothalamic–pituitary–adrenal (HPA) axis, which coordinates the response to stress in conjunction with the sympathetic nervous system (Figure 1). The HPA axis is critical for life and operates by feed‐back loops to regulate glucocorticoid concentrations, thereby maintaining a dynamic and allostatic system homeostasis (Lightman et al., 2020). In addition, brain aldosterone‐preferring MRs regulate salt and water balance, including their associated behaviours (de Kloet & Joëls, 2017).

Corticosteroid signalling becomes dysregulated in certain disease states, or by chronic exposure to synthetic glucocorticoids used in treatment and hormonal replacement strategies, and this dysregulation can enhance the vulnerability to disease (Lightman et al., 2020). Furthermore, MR and GR may contribute to (brain) disease, in conditions that are not typically associated with high hormone levels. In stress‐related pathologies for example, MR antagonists (MRAs) and other pharmacological targeting drugs that block or stimulate the receptor might ameliorate dysregulated MR effects, providing further evidence and a nuanced understanding of MRs role in brain health and disease. This review focusses on the specific role of MR (often in concert with GR) in mediating corticosteroid effects in the brain on gene transcription and wider cellular processes in the control of cognitive and emotional function.

### 1.1 MR pharmacology

**FIGURE 2:**
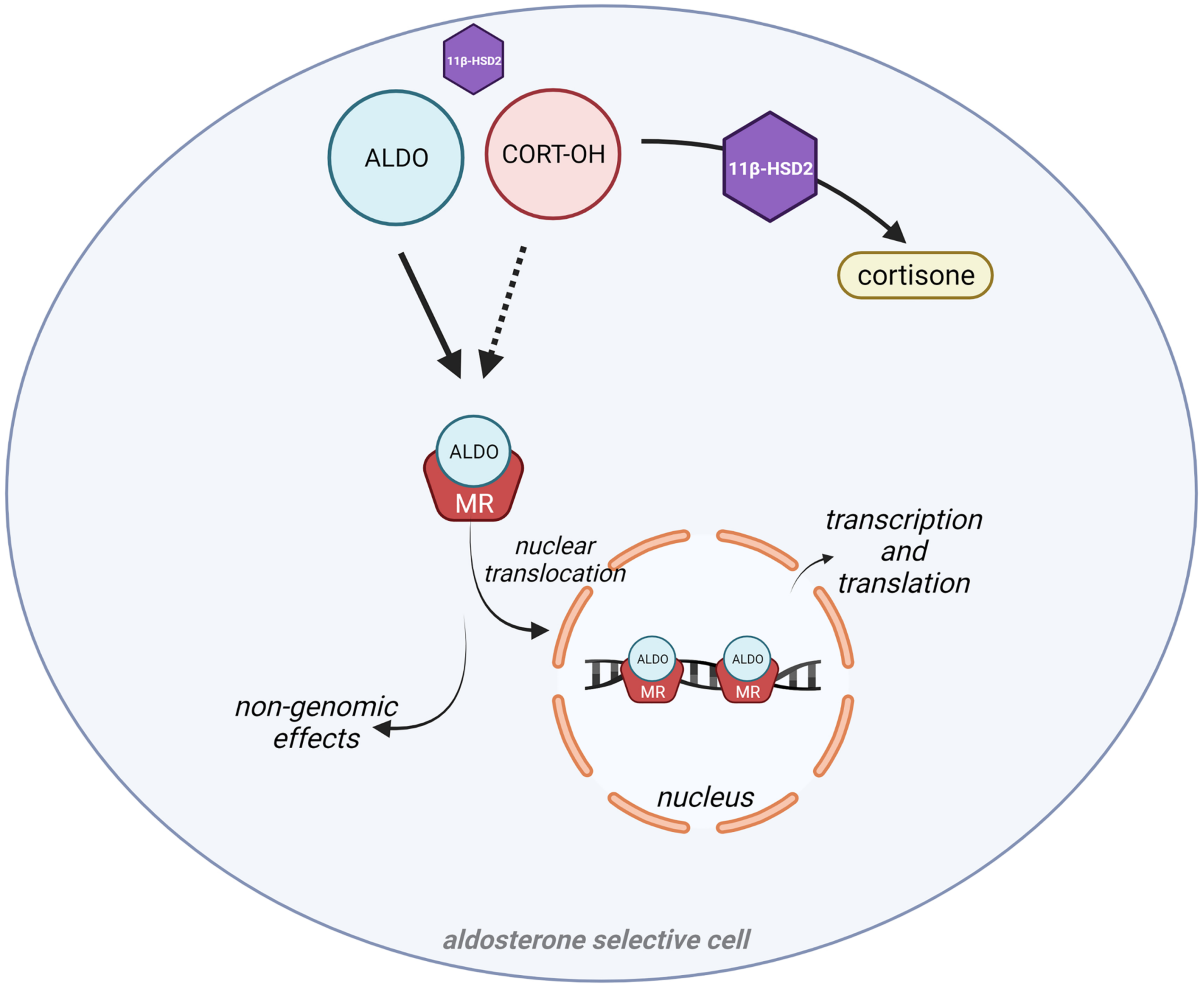
In the blood, cortisol (CORT) circulates at 100‐1000‐fold higher concentrations than aldosterone (ALDO) but binds with equally high affinity to the MR. This CORT:ALDO ratio is more profound in the brain, which in MR‐expressing cells results in CORT occupying MRs and restricting binding by ALDO. However, ALDO selective cells express hydroxysteroid 11‐beta dehydrogenase 2 (11‐βHSD2) which converts CORT into its inactive keto variant cortisone. Upon ligand binding, either rapid non‐genomic MR effects occur or nuclear translocation of MR results in DNA binding at targeted sequences to exert genomic effects

Brain corticosteroid concentrations are influenced by several factors, including brain penetration and local enzymatic conversion in specific brain areas. Foremost, the access of exogenous glucocorticoids and endogenous cortisol to the brain is limited by the expression levels of P‐glycoprotein (Pgp) expression (Karssen et al., 2001), a multidrug transporter in the luminal (blood‐facing) membrane of endothelial cells of the blood brain barrier. Endogenous corticosterone—the sole glucocorticoid in rodents—is not a Pgp substrate, but since no other major differences between cortisol and corticosterone are known, they are collectively abbreviated here as ‘CORT’. The potential effects of corticosteroid hormones on neuronal activity are decided by the distribution of their receptors. In limbic‐frontocortical neurons where MR expression is high (Figure 1), the MR receptor is not protected from CORT by co‐localisation with hydroxysteroid 11‐beta dehydrogenase 2 (11‐βHSD2). Instead, co‐localisation with hydroxysteroid 11‐beta dehydrogenase 1 (11‐βHSD1) reductase, another cytosolic enzyme widely present in neurons and glial cells (Wyrwoll et al., 2011), converts the inactive keto‐variant cortisone (11‐dehydro‐17‐hydroxycorticosterone) to its active state ‘CORT’. Given the approximately 100‐1000 fold excess of CORT over aldosterone, MR ‘sees’ abundant CORT and, as a result, preferential binding by CORT occurs. This outcompeting of aldosterone occurs even though CORT is partially bound in the blood to corticosteroid‐binding globulin (CBG, or transcortin). These MR sites have been termed ‘CORT preferring’, a term used first by John Funder's group in the early 1980s (Krozowski & Funder, 1983) and since widely adopted by various leading researchers in the field (de Kloet et al., 2018). Whether aldosterone also is locally synthesised remains to be confirmed (Gomez‐Sanchez et al., 2005), but aldosterone‐selective MR binding mostly occurs by hydroxysteroid 11‐beta dehydrogenase 2 (11‐βHSD2) inactivation of CORT to its 11‐dehydro metabolites. In the brain, this occurs predominantly in the brain stem nuclei of the solitary tract (NTS) and in discrete subpopulations of hypothalamic neurons (Geerling & Loewy, 2009) (Figures 1 and 2), where these MRs have been called ‘*aldosterone selective*’. The well‐described interactions of MR with GR should be limited to ‘CORT preferring’ MRs, because of the lack of the endogenous GR ligand in 11‐βHSD2‐expressing cells. Ligand‐dependent interactions with transcriptional coregulator proteins may further add to CORT/aldosterone specific effects in a cell‐ and gene‐dependent manner (Fuller et al., 2017).

The high affinity of MR for CORT predicts that receptor expression levels can be limiting for its effects. A 10‐fold higher binding affinity for glucocorticoids means that MR is substantially occupied under basal conditions (de Kloet et al., 2005), as demonstrated in the ultradian rhythm of the HPA axis where MR has extended activation and extended DNA binding duration during the inter‐pulse interval. In contrast, GR activated at the oscillating pulse peak transiently binds to and dissociates from DNA, a phenomenon termed ‘*rapid cycling’*, which tracks the rise and fall of ligand concentration (Stavreva et al., 2009). Functionally, MR activation under basal conditions is in line with a ‘preparative’ role in the stress response, where it determines stressor appraisal and initial reactivity, whereas GR becomes activated with gradual increases in stress hormones and (for example) is involved in the consolidation of stress‐related memories (de Kloet et al., 2018). Of note, because brain access of cortisol is lower than that of corticosterone, predictions on receptor occupancy in the human brain may differ slightly from that observed in laboratory rodents (Karssen et al., 2001).

The high affinity of MR for glucocorticoids is typically interpreted by its genomic effects. However, rapid non‐genomic effects also have been demonstrated for membrane‐associated MR and GR that require 10‐fold higher corticosteroid levels in comparison to their genomic nuclear actions (Joëls & de Kloet, 2017; Nahar et al., 2015). Yet some data indicate that genomic MR‐mediated responses can depend on high CORT levels (Mifsud & Reul, 2016; van Weert et al., 2017), which is in apparent contrast to ligand binding data from early studies (Reul & de Kloet, 1985).

### 1.2 Different MR ligands

The transactivation strength of aldosterone is reportedly more potent than that of endogenous glucocorticoids. Aldosterone dissociates from MR more slowly, and the receptor undergoes several ligand‐receptor conformational changes (Grossmann et al., 2004). Conversely, synthetic glucocorticoids display a very rapid off‐rate from the MR, potentially explaining why potency in vivo is much less than would be anticipated purely from steady‐state ligand binding affinity data (Reul et al., 2000). Other steroidal ligands and their potencies are listed in Table 1.

**TABLE 1:** Ligands of the mineralocorticoid receptor

Compound name	Endogenous ligand	Synthetic ligand	Function type	Receptor binding affinity	Steroidal	Specific
Progesterone	+		Antagonist	IC_50_ 30 nM	+	PR > MR >> GR
Spironolactone		+	Antagonist	IC_50_ 66 nM	+	MR > AR >> PR >> GR
Eplerenone		+	Antagonist	IC_50_ 990 nM	+	MR >>> GR, AR > PR
Esaxerenone		+	Antagonist	IC_50_ 3.7 nM	**−**	MR
Finerenone		+	Antagonist	IC_50_ 18 nM	**−**	MR >>> GR, AR, PR
Fludrocortisone		+	Agonist	IC_50_ 1.01 nM	+	MR > GR

*Note*: The physiological and steroidal status, function activity, receptor binding affinity, specificity for progesterone, spironolactone, eplerenone (Kolkhof & Bärfacker, 2017), esaxerenone (Arai et al., 2015), finerenone (Bärfacker et al., 2012), fludrocortisone (Gesmundo et al., 2016; Grossmann et al., 2004). The estrogen receptor was not included due to insignificant affinity to the named compounds.

### 1.3 MR expression profile in the brain

MR expression extends from polarised epithelial tissues to nonepithelial cells such as hippocampal and hypothalamic neurons, cardiomyocytes, and adipocytes. In the brain, the MR expression profile is typically more restricted than that of GR (Reul & de Kloet, 1985). The aldosterone selective MRs (in 11‐βHSD2 expressing cells) are discretely distributed in the brain, with an abundance in the NTS (Figure 1) to control physiology and behaviour related to sodium balance and sodium transport by epithelial cells (de Kloet et al., 2018; Geerling & Loewy, 2009). A proportion of these NTS neurons project to the parabrachial/locus coeruleus nuclei; some also innervate the ventrolateral bed nucleus of the stria terminalis (BNST), and a small number of NTS neurons project to the ventral tegmental area (VTA), central amygdala and hypothalamus which regulate motivation and arousal, the reward pathways and cognitive functions related to salt balance (de Kloet & Joëls, 2017). These receptors may be important in human psychopathology in relation to Conn's disease and other forms of hyperaldosteronism (discussed later in detail) (Gendreitzig et al., 2021).

Brain areas expressing CORT‐preferring MR include the pre‐frontal cortex, hippocampus, lateral septum thalamic nuclei, hypothalamic nuclei, and medial and central amygdala (Reul & de Kloet, 1985) (Figure 1). MR is typically viewed as neuronal, but single cell mRNA profiling also suggests MR expression in astrocytes (Viho et al., 2022). Hippocampal MR contributes to the indirect negative feedback regulation of the HPA axis and affects processes involved in the control of emotion, cognition, and behaviour (Vogel et al., 2016). Hippocampal MR expression is high throughout all principal glutamatergic cell layers (Reul & de Kloet, 1985) with the highest levels in neurons of the Cornu Ammonis 2 (CA2). Strikingly, from the embryonic stage through to adulthood, the effects of steroid receptor signalling on genetic programming may be directly linked to differentiation via MR‐mediated pathways that determine CA2 identity and CA2‐dependent behaviours (McCann et al., 2021). MR‐directed cell type‐specific molecular signatures involved in cellular processes and disease states in the brain will require further interrogation using advanced techniques such as single cell RNA sequencing (scRNA‐seq). In fact, scRNA‐seq under basal conditions has identified higher expression of MR than GR in GABAergic neurons of the hippocampus (Viho et al., 2022). Models assessing fear extinction and behavioural responses to stress have identified CORT preferring MR projections from infralimbic origin, to innervate the locus coeruleus (LC) and NTS as well as intercalate amygdala neurons that then exert a GABAergic control over the central amygdala (Milad & Quirk, 2012). Indeed, the prelimbic‐ and infralimbic PFC (prefrontal cortex) are involved in both fear expression and extinction (Milad & Quirk, 2012). It will be of interest to establish a more complete picture of mRNA expression via scRNASeq data for MR in the human brain.

### 1.4 MR signalling at the cellular level

MR signalling is diverse, and involves receptor isoforms as well as post‐translational modifications. These modifications lead to specific responses (for further reading on receptor structure and variants, see: Grossmann et al., 2021; ter Heegde et al., 2015). MR signalling generally involves four defined interactions: ligand binding, direct and indirect DNA binding, interdomain interactions, and coregulatory interactions (Poulsen et al., 2018; Rivers et al., 2019), although our mechanistic understanding is incomplete for all four (Fuller et al., 2021). The MR harbours three functional domains: a N‐terminal domain (NTD) with an intrinsically disordered N terminus, a DNA‐binding domain (DBD), and a C‐terminal ligand‐binding domain (LBD) (linked to the DBD via a ‘*hinge region’*; Grossmann et al., 2021). Unliganded MR is mostly cytosolic and within a chaperone HSP90 / FKBP51‐containing multiprotein complex. Ligand (agonist) binding induces changes in this complex and initiates subsequent nuclear translocation of MR (Gallo et al., 2007). Once nuclear, MR dissociates from its chaperone HSP90 to form homo‐ or MR‐GR heterodimers (Liu et al., 1996; Rivers et al., 2019) which then bind to GREs (glucocorticoid response elements) at target genes. In the brain, interactions with GREs seem to predominate over other potential types of interactions with the DNA for both MR and GR (Polman et al., 2013; van Weert et al., 2017). MR and GR (as well as progesterone receptors and androgen receptors) can all bind to GRE sequences, and the nearby binding of other transcription factors can induce specificity as observed with NeuroD transcription proteins and MR (van Weert et al., 2017). Nevertheless, steroid receptors share GRE‐driven targets genes, and MR regulation of FKBP5 abundance is, in fact, a mechanism by which GR sensitivity can be affected (Hartmann et al., 2021).

At the DNA level, specific ligand‐induced conformational changes facilitate the recruitment of coregulatory proteins, and interactions explicit to the cell type, to regulate gene expression (Fuller et al., 2017). Interestingly, aldosterone selectively induces an interaction between the NTD and the C‐terminus (N/C) of the LBD, defining the ligand response (Pippal et al., 2009). MR (and GR) activity differs by varying N‐terminus lengths which result from alternative mRNA translation (Faresse, 2014). Our current understanding of modulation of the above‐described mechanisms, via post‐translational modifications of MR and its interacting partners (Jiménez‐Canino et al., 2017), is limited for the brain despite its likely relevance.

### 1.5 MR‐mediated effects in the brain

MR became the central focus as a moderator of early stress responses, because this corticosteroid receptor was found to mediate non‐genomic rapid and transient effects on neurotransmission and synaptic plasticity in the hippocampus and amygdala after stress (Karst et al., 2010; Treviño & Gorelick, 2021). This was specifically shown with the rapid and transient increase of miniature excitatory postsynaptic currents (mEPSCs) following treatment with CORT, whereby signalling was transduced by MRs located pre‐synaptically at the plasma membrane and regulated the eventual release of glutamate (Groeneweg et al., 2012; Karst et al., 2010). As such, the mEPSC frequency in dorsal hippocampal CA1 neurons follows the (ultradian) CORT pulse amplitude (Sarabdjitsingh et al., 2016). Of note, non‐genomic MR effects can determine subsequent genomic mechanisms, which have been defined as ‘*metaplasticity*’ (Chatterjee & Sikdar, 2014; Karst et al., 2010; Sarabdjitsingh et al., 2014).

#### 1.5.1 MR effects on the peripheral stress response

The stress response maintains homeostasis through autoregulatory neural and hormonal systems and a close association with central and peripheral clocks (Russell & Lightman, 2019). In response to stressors, circuits converge on the brainstem to activate the sympathetic nervous system (Herman et al., 2016), as well as converging on the hypothalamic paraventricular nucleus to stimulate the HPA axis (Lightman et al., 2020). MR also crucially maintains the HPA axis set‐point, as demonstrated in adrenalectomised rats administered with low‐dose corticosteroids (Dallman et al., 1989). HPA axis regulation by MR was further shown when using the MR antagonist RU28318, which increased basal HPA axis activity and potentiated the initial rise in ACTH and CORT secretion in response to stress (Ratka et al., 1989). Furthermore, MR stimulates the sympathetic outflow, as confirmed by denervation studies in rat models of hypertension (Rahmouni et al., 2001). In fact, MR is expressed in rat pre‐autonomic neurons in the hypothalamus and this expression may contribute a role for MR in initiating stress responses (Chen et al., 2014).

#### 1.5.2 MR effects on neurogenesis

Glucocorticoids (GCs) at high concentrations are well defined inhibitors of proliferation of neural stem progenitor cells (NSPCs) and also cause a decrease in adult hippocampal neurogenesis (AHN). These inhibitory actions have been linked to age‐related cognitive impairment (for an extensive review, see: Fitzsimons et al., 2016), as well as being linked to other metabolic and psychiatric diseases (Bornstein et al., 2019). GC oscillations reportedly preserve specific GR‐expressing NSPCs during ageing, preventing their activation via methylation of specific gene promoters and thus preserving a dormant NSPC pool to support the neuroplasticity reserve for the ageing brain (Schouten et al., 2020). In the physiologically complex condition of adrenalectomy, beneficial effects of CORT supplementation are attributed to tonic MR stimulation (Krugers et al., 2007). In a recent study using conditional knockout mice, MR and GR cooperation conferred protection from neurodegeneration in the dentate gyrus (DG), by maintaining adult hippocampal neurogenesis and promoting the survival of DG granule cells (Oakley et al., 2021). MR involvement in neurogenesis and cell fate may affect stress‐related learning and memory deficits and psychiatric disease.

#### 1.5.3 MR effects on neuronal activity in rodent models

Nuclear MR‐mediated effects set the reactivity of neurons to stress‐related stimuli. In hippocampal neurons, this MR activation is required for cell viability and for the stable transfer of excitatory signals, whereas GR‐dependent actions transiently restore a raised excitability through MR signalling (Joëls & de Kloet, 2017). Early studies demonstrated that genomic GR‐mediated effects on excitability are opposite to those by genomic MR in the dorsal hippocampal CA1. For L‐type calcium currents, the absence of CORT produced high amplitudes that gradually reduced with low CORT doses and increased following high CORT application (Diamond et al., 2007; Joels, 2006). Such findings form the basis of considerations on the importance of an MR/GR balance (de Kloet et al., 2018). This outcome differs in the amygdala where rapid MR‐dependent increases in excitability occurs, by cooperation with nuclear GR, and is prolonged with noradrenaline exposure (as seen in stress; Karst & Joëls, 2016).

#### 1.5.4 MR effects on cognitive and emotional function

A number of pharmacological studies have described MR‐regulated pathways in regulating emotional and cognitive reactivity, by affecting the appraisal of novel situations, learning and search‐escape strategies, and behavioural response selection. Enhanced MR expression induces a switch from hippocampal to dorsal striatal based habit learning, and facilitates this learning shift to guide behaviour under stress (Joëls & de Kloet, 2017). In genetically modified MR‐deficient models, learning and memory performance and behavioural adaptation were reduced in strategic contexts (Berger et al., 2006; Joëls & de Kloet, 2017). These adaptations were improved by overexpressing MR (Lai et al., 2007; Mitra et al., 2009; Rozeboom et al., 2007) and when combined with decreased GR expression, improved spatial memory as well as behavioural flexibility (Harris et al., 2013).

MR function may also modify the effects of early life stress (ELS). In mice exposed to low stress conditions, forebrain MR overexpression alleviated ELS‐driven stress effects, as assessed by contextual learning, compared with wild type. ELS effects on hippocampal neurogenesis were determined by increases in doublecortin (DCX, a microtubule‐stabilising protein) positive cells in the granular cell layers of the hippocampal DG in control mice. ELS effects (although not significant) were observed on synaptic transmission, regeneration, and DG cell maturation (Kanatsou et al., 2017). The interpretation of interactions between MR expression and effects of ELS is challenging in relation to the time at which MR is required. Whether these effects reverse injury or prevent delayed effects remains to be determined.

To summarise, preclinical research *in vivo* and *in vitro* has developed our understanding of the MRs functional role in the brain and indicates a crucial role whereby MR is involved in the initial response to stress and also may confer protective effects on brain function, particularly for chronic or adverse early life stress.

### 1.6 Role of MR in cognitive and emotional functions in humans: healthy individuals

Much of our understanding of MR function on HPA axis, CORT, and cognition in animal studies (Joëls et al., 2008) has proven to be transferable to humans. Because MRs are expressed abundantly in the limbic circuitry, researchers have studied their regulatory role in depression and cognitive dysfunction in humans (Otte, Wingenfeld, Kuehl, Kaczmarczyk, et al., 2015). Here, we focus on studies that have examined MR effects on cognitive and emotional function in healthy individuals and patients with stress‐associated disorders.

The effects of CORT on cognition per se, such as memory and executive function of healthy individuals, have been investigated intensively (Shields et al., 2017). Increased CORT concentrations have been shown to consistently enhance memory consolidation but impair memory retrieval (Wolf, 2017). Most of these experimental studies administered CORT, or used psychosocial stress exposure, which led to the stimulation of both GR and MR. More specific pharmacological MR targeting has been used to block or stimulate receptor function, by the single administration of spironolactone (antagonist) or fludrocortisone (agonist), respectively (Wingenfeld & Otte, 2019).

#### 1.6.1 MR blockade

Studies have demonstrated that blocking MR impairs memory retrieval, including verbal, autobiographic, and visuo‐spatial memory (Otte et al., 2007; Rimmele et al., 2013; Young et al., 2016), thus highlighting MR‐regulated pathways in hippocampal‐based memory. However, learning and working memory were not affected by MR blockade (Cornelisse et al., 2011; Otte et al., 2007; Young et al., 2016). Non‐hippocampal cognitive processes, such as selective attention and psychomotor speed, also were unaffected by spironolactone (Cornelisse et al., 2011; Otte et al., 2007). Thus, intact hippocampal MR activation appears important for optimal human memory regulation. However, MR blockade increases CORT concentrations, potentially shifting MR/GR balance towards more GR activation, and so GR involvement also must be considered in these effects.

Another line of research investigated the role of MR after psychosocial stress. Interestingly, spironolactone treatment prevented stress‐induced CORT effects on response inhibition, stimulus–response learning, and delay conditioning (Vogel et al., 2016; Vogel et al., 2017), including cognitive function beyond hippocampus‐dependent processes. At the neuroimaging level, stress is associated with a reduced activation of the hippocampus, and an enhanced amygdala activation and amygdala‐striatal connectivity, which were found to depend on MR availability (Vogel et al., 2016; Vogel et al., 2017). These studies emphasise the importance of amygdalae MR, in line with the observation that MR blockade was most effective for emotional stimuli (Rimmele et al., 2013).

In summary, MR blockade has mostly impairing effects on cognition in healthy individuals, which fits closely with the animal data. Of note, the observed effects reflect a one‐time administration of the MR antagonist, whereas the effects of chronic or repeated MR blockade have been less well investigated. Importantly, one study treated obese individuals with low‐dose spironolactone over 6 weeks and found improved rather than impaired learning performance (Rotenstein et al., 2015). This may be relevant in situations such as obesity, which is associated with increased MR activity in peripheral tissues and possibly in central tissues as well (Infante et al., 2017).

#### 1.6.2 MR stimulation

Given that MR antagonism impairs cognitive performance, MR activation also may improve it. Indeed, several studies showed a single administration of fludrocortisone acutely enhances verbal learning performance and visuo‐spatial memory retrieval but did not enhance autobiographic memory retrieval. Notably, visuo‐spatial memory is strongly hippocampus‐dependent, and the effects of fludrocortisone are likely to be explained by high MR density in this brain region (Wingenfeld & Otte, 2019).

The data are less clear regarding other cognitive domains, as reviewed elsewhere (Wingenfeld & Otte, 2019). In brief, no effects of MR activation were found with respect to executive function (Groch et al., 2013; Otte, Wingenfeld, Kuehl, Kaczmarczyk, et al., 2015), but young healthy humans made riskier decisions after fludrocortisone administration compared with placebo treatment (Deuter et al., 2017). Furthermore, an enhanced attentional bias towards negative faces was observed after fludrocortisone (Schultebraucks et al., 2016), although this bias was not replicable in a more heterogeneous sample of facial images (Nowacki et al., 2021). These findings are compatible with the hypothesis that MR activation stimulates the brain salience network, indicating an involvement in appraisal of novel situations, in attentional vigilance to salient information, and in behavioural flexibility (Vogel et al., 2016).

The salience network also is important for social cognition. Humans must perform complex social tasks while stressed, challenged, or when emotionally aroused. Accordingly, several studies demonstrate that psychosocial stress can enhance several aspects of social cognition and prosocial behaviours (von Dawans et al., 2012; Wolf, 2017). Additionally, MR stimulation was shown to increase empathy scores (Nowacki, Wingenfeld, Kaczmarczyk, Chae, Abu‐Tir, et al., 2020a; Wingenfeld et al., 2014), adding to a role for MR in adequate and successful social functioning (Wingenfeld & Otte, 2019).

#### 1.6.3 Considerations

Pharmacological MR manipulation comes with some considerations. Fludrocortisone can bind to GR (Grossmann et al., 2004) and also can reduce CORT release (Nowacki, Wingenfeld, Kaczmarczyk, Chae, Abu‐Tir, et al., 2020a), highlighting MR function in HPA axis feedback regulation, but impacting on GR activation and thus complicating the interpretation of results. Fludrocortisone activates aldosterone‐selective MR in the NTS, although no effects were found on blood pressure (Wingenfeld & Otte, 2019) or on aldosterone release (Nowacki, Wingenfeld, Kaczmarczyk, Chae, Salchow, et al., 2020b). Spironolactone is known to have effects on progesterone receptors (Quinkler & Diederich, 2002) and, in view of progesterone binding to the MR, sex‐specific effects on MR function and cognition deserve more attention. Although some studies report no sex effects (Deuter et al., 2017; Piber et al., 2016), the consequence of genetic MR haplotypes did critically depend on sex (Klok, Vreeburg, et al., 2011c; Wirz et al., 2017). Notably, age‐related differences were reported in fludrocortisone effects in major depressive disorder (MDD) patients (see below Otte, Wingenfeld, Kuehl, Kaczmarczyk, et al., 2015; Otte, Wingenfeld, Kuehl, Richter, et al., 2015) compared with controls (Hinkelmann et al., 2015).

Overall, evidence is growing for MR function as essential in cognition and emotional function. These MR‐mediated effects seem to be moderated by age, sex, and potentially other variables. Thus, further research into MR effects is warranted not only in healthy humans but also in patients with stress‐related mental disorders who often show alterations in cognitive and emotional function.

### 1.7 Mineralocorticoid receptors in major depressive disorder (MDD) and borderline personality disorder (BPD)

Several mental disorders are characterised by HPA axis dysfunction. In this review, we specifically focus on MDD and BPD as two examples of stress‐associated disorders. Historically, research on corticosteroid receptor function in mental disorders has predominantly focussed on GR alterations (Holsboer & Ising, 2009). However, variations in the MR gene are commonly associated with negative memory bias, amygdala reactivity, and life adversity in mental disorders (Bogdan et al., 2012; Vinkers et al., 2015; Vogel et al., 2016). Additionally, altered aldosterone concentrations have been repeatedly found in MDD (Nowacki, Wingenfeld, Kaczmarczyk, Chae, Salchow, et al., 2020b). Therefore, an association is plausible between the MR function with cognitive and emotional dysfunction. Given the differences in localisation of aldosterone‐selective and CORT‐preferring MR, pathophysiological mechanisms should differ depending on the ligands acting on MR.

#### 1.7.1 Major depressive disorder

MDD is one of the most prevalent mental disorders (Otte et al., 2016). A major depressive episode has depressed mood, and loss of interest or pleasure, as its core symptoms. Cognitive deficits, such as memory and attention, also feature and some studies showed an association between elevated CORT and cognitive impairments (Gomez et al., 2006). Endocrine features often include increased basal CORT release, reduced feedback sensitivity, and changes in GR function (Holsboer & Ising, 2009; Otte et al., 2016). In addition to elevated aldosterone levels in patients (Nowacki, Wingenfeld, Kaczmarczyk, Chae, Salchow, et al., 2020b), decreased MR expression was observed in the post mortem hippocampus and prefrontal cortex (Klok, Alt, et al., 2011a; Medina et al., 2013; Qi et al., 2013). Furthermore, MR dysregulation with aberrant DNA methylation were reported in treatment‐resistant depression, in gestational pregnancy, and in adolescence (Galbally et al., 2020; Stonawski et al., 2019).

**FIGURE 3:**
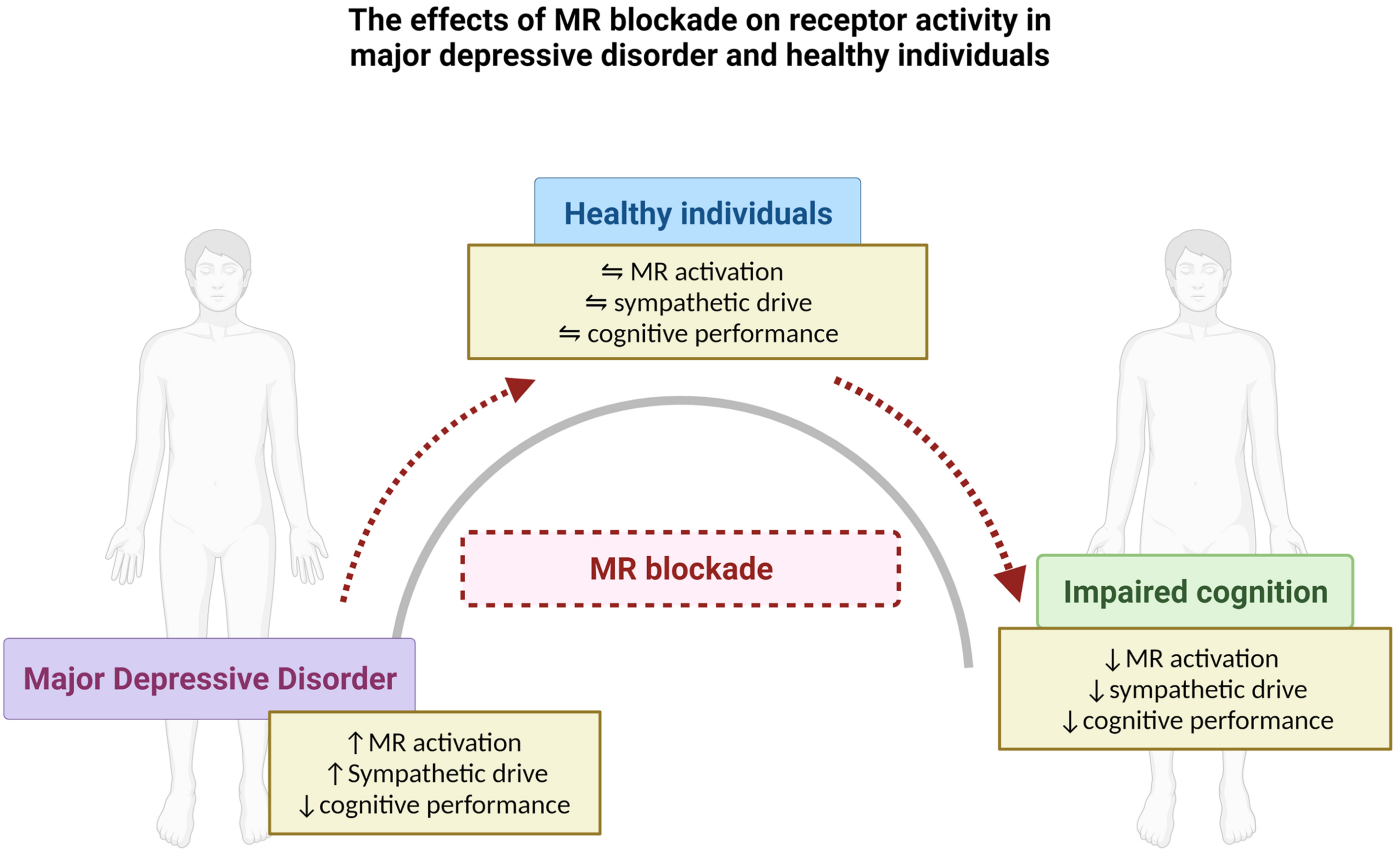
Hypothetical model of MR activity in patients with major depressive disorder and healthy individuals and the potential effects of MR blockade. Sympathetic activity, cognitive performance such as memory, concentration and attention, executive function, as well as mood, follow an inverted U‐curve by level of MR activation

Haplotypes of single nucleotide polymorphisms (SNPs) of the MR gene (NR3C2) have been associated with depression (Klok, Giltay, et al., 2011b), with stress‐induced reward related learning deficits (Bogdan et al., 2010), and with cognitive function (Keller et al., 2017). An association between MR polymorphisms and emotional memory has been reported in remitted MDD patients (Vrijsen et al., 2015). Interestingly, this association was more prevalent in patients with a history of childhood trauma, which fits with an earlier study in adolescents (Bogdan et al., 2012), and with links to animal studies on MR overexpression in mice (Kanatsou et al., 2017). MR haplotypes even were shown to sex‐dependently moderate a susceptibility to depression following childhood maltreatment, where females were advantaged by the CA haplotype and males were advantaged with the GA and CG haplotypes (Vinkers et al., 2015). In general, the gain‐of‐function haplotype (CA), by MR‐2G/C and MRI180V, has a protective effect in all these aspects (Vinkers et al., 2015) and, together with discussions from animal studies, highlights the relevance of identifying factors contributing to resilience and gender differences (Bonapersona et al., 2019).

nterestingly, MR blockade appeared to ‘normalise’ cognitive empathy in MDD patients. Cognitive empathy scores were higher in patients with MDD compared with controls in the placebo condition, but not after single administration of spironolactone (Wingenfeld et al., 2016). Together, the finding in relation to MDD fits with the model presented in Figure 3, hypothesising that enhanced MR signalling due to increased CORT secretion might be responsible for some aspects of impaired cognition in patients. Therefore, future studies should investigate whether treatment with MR antagonists can exert beneficial effects on cognitive symptoms in MDD.

Several studies have demonstrated acute beneficial effects of MR stimulation on verbal learning performance and visuo‐spatial memory retrieval in healthy humans (Groch et al., 2013; Hinkelmann et al., 2015; Piber et al., 2016), thus making it plausible that MR stimulation with fludrocortisone could improve aspects of cognitive performance in MDD. A dedicated study found promising initial results, where relatively young medication‐free patients with MDD as well as healthy control individuals showed improved verbal memory and executive function after one‐time administration of fludrocortisone compared with placebo (Otte, Wingenfeld, Kuehl, Richter, et al., 2015). However, a recent study tested if such effects could be extended to depression‐related emotionally salient stimuli, but there was no effect of fludrocortisone on selective attention to emotional stimuli or on facial emotion recognition in MDD and healthy controls (Nowacki et al., 2021).

It is striking that MR blockade, as well as its stimulation, might have beneficial effects on cognition in MDD. Although MR blockade seems to affect social cognition, MR stimulation seems to enhance especially non‐emotional or more basic cognitive functions such as verbal memory and executive function. However, there are too few studies to draw final conclusions. In summary, what is known of acute MR effects in MDD differ depending on the cognitive domain that is examined. Age and sex seem to be important moderators in this respect (Hinkelmann et al., 2015; Otte, Wingenfeld, Kuehl, Kaczmarczyk, et al., 2015). Finally, the MDD population is heterogenous with melancholic, psychotic, or anxious subtypes. Some evidence suggests that MR alterations are most pronounced in patients with psychotic symptoms or with treatment resistance (Juruena et al., 2013; Lembke et al., 2013).

In terms of biomarkers, aldosterone and MR function have been related to depression in a study by Büttner et al. (2015). These authors identified biomarkers for both peripheral MR‐underactivity and central MR‐overactivity predicted refractoriness. This predicted depression treatment response to standard antidepressants was observed in an in‐patient cohort and, more consistently, in males (Büttner et al., 2015).

#### 1.7.2 Borderline personality disorder

Borderline personality disorder (BPD) is characterised by intense and rapidly changing mood states and impulsivity, self‐injurious behaviours, fear of abandonment, unstable relationships, and unstable self‐image. Although a consistent pattern of neuropsychological deficits has not be found in patients with BPD, there is some evidence for difficulties in social cognition, especially mentalisation (Bora, 2021). Early life adversities are highly prevalent, and childhood trauma is a major antecedent for BPD. Several studies observed alterations in the HPA axis in patients with BPD (Wingenfeld & Otte, 2019), but MR function is mostly neglected in these studies. In a small study, MR and GR sensitivity did not differ between BPD patients and controls (Fischer et al., 2014). Fludrocortisone treatment led to improved working memory performance in BPD patients, but verbal and visuospatial memory were impaired in BPD women compared with controls (Wingenfeld & Otte, 2019). Interestingly, an earlier study found that GR and MR activation by hydrocortisone enhanced memory retrieval in BPD patients (Wingenfeld et al., 2013). In summary, differences between patients and healthy individuals only were seen in hippocampus‐associated cognitive domains.

BPD symptoms are exacerbated by stressful interpersonal events and deteriorates social cognition and emotional empathy scores (Lazarus et al., 2014; Wingenfeld et al., 2018). Similar to healthy controls, patients had higher emotional empathy scores after MR stimulation (Wingenfeld et al., 2014). The finding of reduced emotional empathy after stress can be interpreted as a stress‐induced ‘fight‐or‐flight’ response in BPD patients, leading to symptoms such as increased impulsivity rather than prosocial behaviours observed in healthy individuals. MR‐regulated pathways in stress effects on social cognition might be of high clinical relevance in BPD.

### 1.8 Endocrine disease and glucocorticoid treatment: Cognitive and emotional function

Given the central role that MR and GR play in cognitive and emotional processes as outlined above, it is not surprising that endocrine diseases and long‐term steroid treatment are associated with alterations in these processes. Although it is often difficult to exactly disentangle MR‐ versus GR‐mediated effects, it is increasingly becoming clear that MR has a prominent role in these effects.

#### 1.8.1 Primary aldosteronism

Primary aldosteronism (PA), or Conn's disease, is characterised by autonomous hypersecretion of aldosterone, either due to an aldosterone‐producing adenoma or due to bilateral adrenal hyperplasia. A systematic review, which included 15 studies (Velema et al., 2017), demonstrated that untreated patients with PA showed lower physical and mental quality of life compared with the general population independent of the cause of PA. Furthermore, PA patients exhibited more psychopathologies including depressive symptoms, anxiety, agitation, and sleep problems. It is likely that overstimulation by aldosterone of central MR contributes to psychopathology in PA (Engler et al., 2019; Künzel, 2012). In fact, a recent study demonstrated that adrenalectomy in patients with PA normalised aldosterone secretion, which then was associated with improved depressive symptoms (Murck et al., 2021). To our knowledge, no study has specifically examined cognitive function in patients with PA versus a matched healthy control group.

#### 1.8.2 Adrenal insufficiency

Adrenal insufficiency (AI), characterised by insufficient glucocorticoid and mineralocorticoid secretion, requires hormone replacement therapy with hydrocortisone and fludrocortisone, respectively. Several reviews concluded that AI patients exhibited impaired physical and mental health and increased psychopathology independent of AI aetiology (Hahner et al., 2021). Importantly, psychopathology, cognition, and quality‐of‐life depended on hydrocortisone treatment regimens that closely mimicked physiological CORT secretion and on sufficient MR activation by fludrocortisone (Schultebraucks et al., 2016). The interdependent contributions of MR and GR to these psychopathological symptoms remain undefined.

#### 1.8.3 Cushing's syndrome

The process of separating MR‐ from GR‐mediated effects is very challenging in Cushing's syndrome (CS), which is characterised by excess glucocorticoid concentrations in circulation, most commonly from chronic glucocorticoid therapy. Aside from a plethora of well‐known metabolic and cardiovascular complications, patients with CS experience several neuropsychiatric sequelae, including memory impairment, cognitive deficits and mood disorders, and a predisposition to anxiety and depression (Piasecka et al., 2020). Brain atrophy from excessive CORT secretion has been repeatedly described in CS. Considering the role of MR in emotional and cognitive functions, and in neuronal integrity, these alterations may be mediated in part by MR. The GR antagonism by mifepristone, which is used clinically to antagonise GR in CS, may shift the MR/GR balance towards MR function, and this shift may contribute to improving depressive symptoms, cognitive function, and metabolic/cardiovascular risk factors (Howland, 2013).

#### 1.8.4 Glucocorticoid treatment

The need for safer and more effective glucocorticoid replacement therapies is paramount. Despite the success of glucocorticoids in the clinic, standard glucocorticoid replacement therapies reduce health‐related quality‐of‐life, for example through adverse metabolic and cardiovascular risk profiles and via neuropsychiatric side effects (Judd et al., 2014; Laugesen et al., 2021). Given the importance of GR and MR cooperation under normal conditions, the adverse psychological, behavioural, and cognitive effects of synthetic glucocorticoids homeostasis may involve a GR and MR imbalance (Judd et al., 2014; Meijer & de Kloet, 2017).

Dexamethasone (DEX) is an example of a potent synthetic glucocorticoids that is highly selective for GR *in vivo* (Reul et al., 2000). The strong activation of GR by DEX causes suppression of HPA axis activity, a reduction in CORT levels and consequently depleting MR of its ligand. As such, this increase and decrease in GR and MR activity, respectively, disturbs system homeostasis regulated by these two receptors (Meijer & de Kloet, 2017) and further supports a role for MR in mood states. An early case report supports this notion (Seifritz et al., 1994). Based on this assumption, it was examined whether add‐on hydrocortisone would ameliorate cognitive and emotional side effects of DEX therapy as part of leukaemia treatment in children. As hypothesised, hydrocortisone attenuated the DEX‐induced mental side effects in those individuals that suffered most strongly (Warris et al., 2016).

### 1.9 Future perspectives

The role of MR in the initial physiological and behavioural reactions to novel circumstances or stressors is crucial for an adaptive stress response (de Kloet & Joëls, 2017; Vogel et al., 2016) and crucial for dynamic and allostatic system homeostasis by the HPA axis (Lightman et al., 2020). There is increasing evidence for an important role of MR function in establishing an effective neuroendocrine response, in signalling cascades underlying resilience, in the development of psychiatric disease (de Kloet et al., 2005) and even for brain function in hypertensives (Brocca et al., 2017). Two other remaining aspects of MR function merit mentioning and have not been discussed. First, acute antagonism of membrane‐bound MR on nociceptive and peripheral neurons produces non‐genomic antinociceptive effects (Shaqura et al., 2016). Second, perhaps best studied non‐genomic MR‐mediated effects are in the vasculature via aldosterone‐activated MR pathways and its interplay with striatin. MR‐STRN3 (striatin 3) interactions (Coutinho et al., 2014) are possibly relevant for key cellular processes in the brain, but regulation of brain vasculature per se may also be important for brain function (Ruhs et al., 2017).

With regards to circuits involved in salt‐seeking behaviours, cognition and emotion, the aldosterone selective MR neurons in the NTS also innervate neurons that express CORT‐preferring MRs in the forebrain and mesolimbic cortical dopaminergic pathways of the VTA (de Kloet & Joëls, 2017). Overall, CORT and aldosterone mediate their effects by targeting cell‐ and region‐specific brain MRs, and yet our understanding of aldosterone effects in the brain undoubtedly remains hindered by experimental challenges with the NTS and other brain regions.

MR‐mediated effects depend on age, gender, genetic variations, and environmental influences. Most MR studies are performed in males, but the antagonism of MR by progesterone blunts MR function and causes inappropriate HPA axis activation (Carey et al., 1995). This is relevant to the menstrual cycle, pregnancy and contraceptives, and may play a role in the increased incidence of depression and anxiety in women. Chronic stress or certain (endocrine) diseases can dysregulate MR activity causing adverse effects on affective state, learning, and memory, as well as behavioural function in susceptible individuals. Considering the important role MR plays in cognition and emotional function in health and disease, MR modulation by pharmacological intervention could relieve stress‐ and endocrine‐related symptoms.

Developing the molecular mechanistic underpinnings of brain MR and teasing out cell and context specificity will further define MR biology, by ascertaining the genomic interactions with its GR companion and other transcription factors, coregulatory proteins, and rapid membrane‐mediated actions (van Weert et al., 2017). Extensive prediction of cell‐specific MR transcriptional partners should be identified using mass spectrometry‐based approaches such as rapid immunoprecipitation of endogenous proteins (RIME) (Papachristou et al., 2018). Likely, these interactions will depend on the specific DNA locus (Meijer et al., 2019) and this may be better understood by combining genome‐wide MR occupancy with that of different coregulators. Finally, MR variants, as reported with the haplotype 2′ (CA) mediated protective action (Hamstra et al., 2017; Klok, Giltay, et al., 2011b; Vinkers et al., 2015) and epigenetic modifications will require further attention and expansion by researchers, to delineate the mechanisms involved in adaptation and resilience.

### 1.10 Nomenclature of targets and ligands

The concise guide to Pharmacology 2021/2022: Nuclear hormone receptors (Alexander et al., 2021) and the http://www.guidetopharmacology.org have been referred to for the correct nomenclature of main protein targets and ligands of the mineralocorticoid receptor (MR).

## AUTHOR CONTRIBUTIONS

SNP: literature search and analysis; writing ‐ original draft preparation; writing ‐ review and editing; preparation of figures. OCM: literature search and analysis; writing ‐ original draft preparation; writing ‐ review and editing; preparation of figures. KW: literature search and analysis; writing ‐ review and editing; preparation of figures. CO: literature search and analysis; writing ‐ review and editing; preparation of figures.

## CONFLICT OF INTEREST

OM's research is financially supported by Corcept Therapeutics, Menlo Park, CA.

## DECLARATION OF TRANSPARENCY AND SCIENTIFIC RIGOUR

This Declaration acknowledges that this paper adheres to the principles for transparent reporting and scientific rigour of preclinical research as stated in the BJP guidelines for Design and Analysis, and as recommended by funding agencies, publishers and other organisations engaged with supporting research.

## Data Availability

No new data have been generated.
